# Esophago-tracheobronchial fistula following treatment of anlotinib in advanced squamous cell lung cancer: Two case reports

**DOI:** 10.1097/MD.0000000000017700

**Published:** 2019-11-01

**Authors:** Pin-Liang Zhang, Zeng-Jun Liu

**Affiliations:** Internal Medicine Department, Shandong Cancer Hospital and Institute, Shandong First Medical University and Shandong Academy of Medical Sciences, Jinan, Shandong Province, P.R. China.

**Keywords:** anlotinib, esophago-tracheobronchial fistula, squamous cell lung cancer

## Abstract

**Rationale:**

Anlotinib, a novel orally administered multitargeted tyrosine kinase inhibitor, inhibiting tumor angiogenesis and growth, significantly prolonged overall survival, and progression-free survival with a manageable safety profile as a third-line therapy among refractory advanced nonsmall cell lung cancer (NSCLC) patients in ALTER 0303 trail (NCT02388919).

**Patient concerns:**

Two squamous cell lung cancer patients with mediastinal metastasis undergoing the treatment of anlotinib developed clinical symptom of cough, which was worse upon ingestion.

**Diagnoses:**

On the basis of patients’ clinical symptoms and radiographic findings, they were diagnosed with acquired esophago-tracheobronchial fistula.

**Interventions:**

We treated them with fully covered self-expandable metallic stents.

**Outcomes:**

The clinical symptom of cough was immediately relieved after palliative treatment. Both patients elected to discontinue anlotinib treatment.

**Lessons:**

These 2 cases imply that a close follow-up schedule for esophago-tracheobronchial fistula should be established when squamous cell lung cancer patients with mediastinal metastasis are undergoing anlotinib therapy. Early detection and adequate treatment are essential for patient symptom relief and survival.

## 1 Introduction

Anlotinib has evolved as standard third-line treatment in Chinese advanced nonsmall cell lung cancer (NSCLC) patients. However, grade 3 or 4 treatment-related adverse events occur in about 20% of patients, and dose reduction or treatment discontinuation is usually required.^[1,2]^ The central type squamous cell lung cancer is a contraindication to anlotinib due to an increased risk of hemoptysis. While the safety of anlotinib therapy for patients with mediastinal metastasis remains unclear. Here, we present 2 cases of squamous cell lung cancer patients with mediastinal metastasis developed esophago-tracheobronchial fistula during anlotinib therapy. Both cases were diagnosed early and treated in a timely manner.

## 2 Case presentation

### 2.1 Case 1

A 55-year-old nonsmoking Chinese woman presented to the local hospital with persistent dry cough and progressive breathlessness. She reported no systemic disease. A computed tomography (CT) scan of the chest revealed a mass (64 × 47 mm) in the lingual segment of upper lobe of the left lung associated with mediastinum and left lower lobar bronchus invasion, along with massive pericardial effusion and mediastinal lymphadenopathy (short axis >15 mm). CT-guided biopsy of the lung mass provided a histopathological diagnosis of squamous cell lung cancer. In the pericardial effusion, cancer cell was reported. On further staging, no other distant metastasis was detected. She accepted 6 cycles of systemic chemotherapy consisting of cisplatin and paclitaxel liposome. A partial oncologic response was confirmed at 1-month follow-up according to RECIST 1.1 criteria (Response Evaluation Criteria in Solid Tumors).

**Figure 1:**
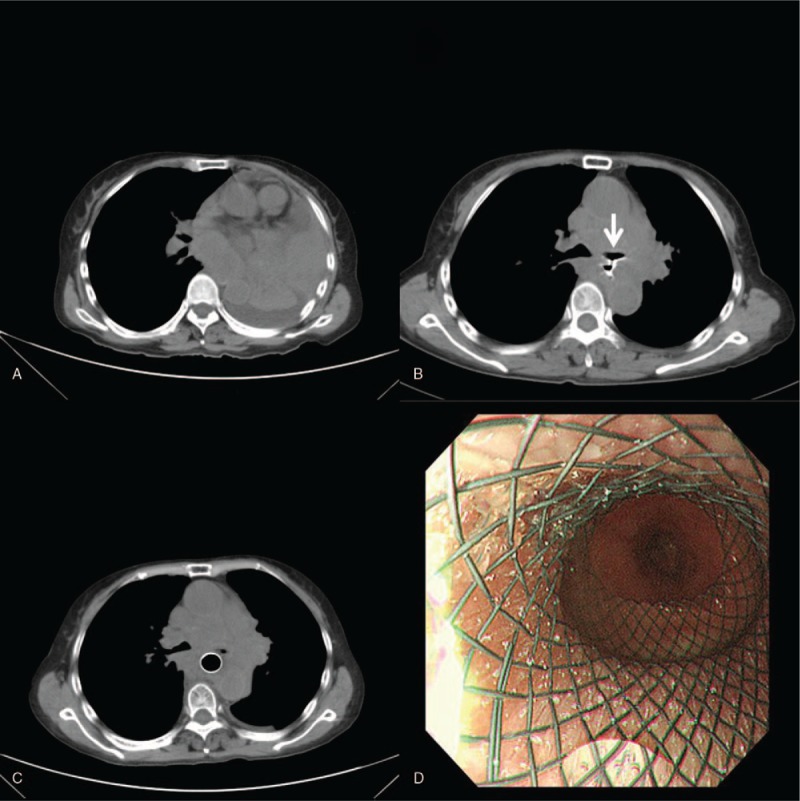
Chest computed tomography (CT) scan showed mediastinal and left hilar mass, along with left total lung atelectasis (A). Left total lung atelectasis disappeared, however, acquired esophago-tracheobronchial fistula (thin arrow) occurred after anlotinib treatment (B). A fully covered self-expandable metallic stent was placed immediately after the diagnosis (C,D).

Almost 3 months later, she came to our department with the main complaint of shortness of breath and difficulty swallowing. A new chest CT scan showed mediastinal and left hilar mass, along with left total lung atelectasis (Fig. 1A). The patient was treated with anlotinib orally, once daily (12 mg) on day 1 to 14 of a 21-day cycle. Just 1 week later, complete remission of dyspnea and lung atelectasis was observed in follow-up visits. One month later, she complained of a cough that became more severe after swallowing. An esophagram after oral ingestion of compound meglumine diatrizoate injection was subsequently performed and revealed a fistula at the mid-esophagus and the left main bronchus. Chest CT imaging showed a small, linear, walled air connection between the left main bronchus and her esophagus, and no pneumonia or pleural effusion was noted (Fig. 1B). Based on above imaging findings, the patient underwent esophagoscopy, revealing a linear defect of her esophageal wall, meanwhile, a fully covered self-expandable metallic stent was placed (Fig. 1C and D). The clinical symptom of cough was immediately relieved after palliative treatment. The patient then elected to discontinue anlotinib treatment. She died of distant metastasis 3 months later, without occurrence of stent migration, bleeding, or secondary fistula.

### 2.2 Case 2

A 53-year-old nonsmoking Chinese man complaining of progressively increasing cough, expectoration, hemoptysis, and dyspnea was referred to a local general hospital. He reported no systemic disease. A contrast-enhanced CT scan of the chest revealed a mass and atelectasis in the lower lobe of right lung, along with mediastinal lymphadenopathy (short axis >15 mm). The bronchoscopy showed an endobronchial lesion in the right main stem, upper and *middle lobar* bronchus, and an obstruction of the *lower lobar* bronchus. The endobronchial biopsy confirmed a diagnosis of squamous cell lung cancer. No distant metastasis was detected in the further staging evaluation. He received 6 chemotherapy cycles with nedaplatin−gemcitabine and 45 Gy (1.8 Gy × 25 fractions) sequential radiation therapy on the lung mass and mediastinal lymph node metastasis.

**Figure 2:**
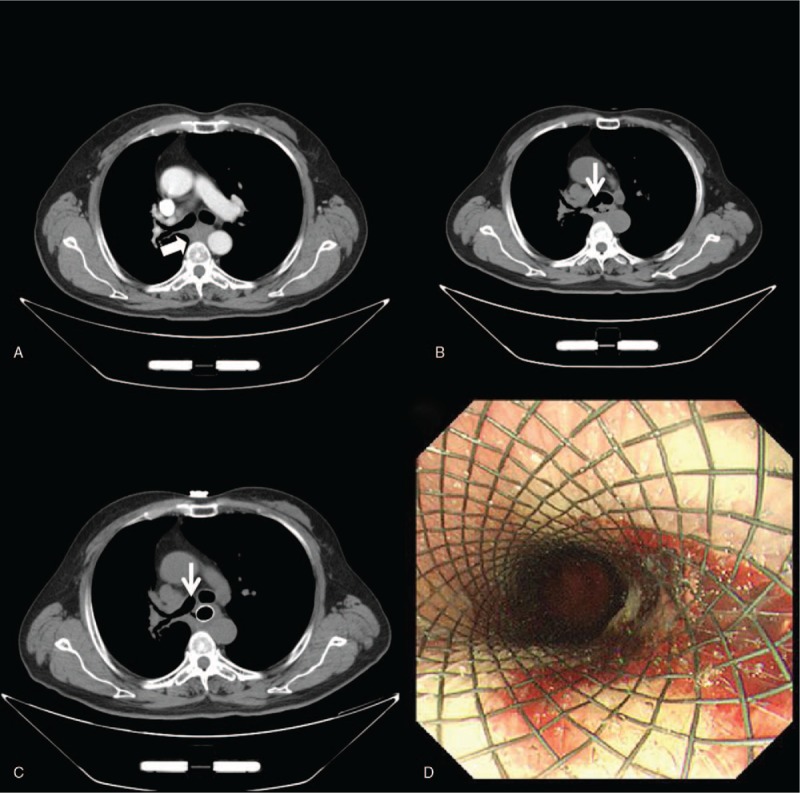
Chest computed tomography (CT) scan showed subcarinal lymph node metastasis (thick arrow) shrunk greatly after anlotinib treatment, however, acquired esophago-tracheobronchial fistula (thin arrow) occurred (B,C). A fully covered self-expandable metallic stent was placed immediately after the diagnosis (C,D).

One year and 5 months later, the patient was referred to our department because of multiple lung metastases. He received 4 cycles of chemotherapy with nedaplatin plus nanoparticle albumin-bound paclitaxel, and a partial response was observed after 2 cycles of chemotherapy. Unfortunately, after 2 months follow-up, a progressive response was confirmed in CT scan evaluation. The patient was then treated with anlotinib orally, once daily (12 mg) on day 1 to 14 of a 21-day cycle. One month later, he complained of a cough that became more severe after swallowing. Chest CT imaging showed a small air connection between the right main bronchus and his esophagus (Fig. 2B). The patient underwent esophagoscopy, revealing a linear defect of his esophageal wall, meanwhile, a fully covered self-expandable metallic stent was placed (Fig. 2C and D). The clinical symptom of cough was immediately relieved. Then the patient elected to discontinue anlotinib treatment. He died of lung cancer progression 6 months later, without occurrence of stent migration, bleeding, or secondary fistula.

## 3 Discussion and conclusion

Anlotinib is a new, orally administered multitargeted tyrosine kinase inhibitor, inhibiting tumor angiogenesis and growth.^[3]^ At a once-daily dose of 12 mg, anlotinib displayed manageable toxicity, a long circulation time, and broad-spectrum antitumor potential.^[4]^ ALTER0303, a randomized double-blind controlled phase 3 trial, examined the efficacy and safety of anlotinib as third-line therapy among Chinese NSCLC patients. Overall survival was significantly improved with a manageable safety profile.^[1]^ Hence, anlotinib was approved by China Food and Drug Administration as a candidate for the third-line treatment in advanced NSCLC on May 9, 2018. The following adverse events are frequently observed during anlotinib treatment: hypertension, elevated thyroid-stimulating hormone, hand and foot syndrome, elevated thyroglobulin, elevated total cholesterol, and diarrhea.^[1,2]^ Among patients with squamous cell lung cancer, hypertension, hyponatremia, and hemoptysis are the most common anlotinib-related adverse events.^[5]^

However, to the best of our knowledge, esophago-tracheobronchial fistula following anlotinib treatment in squamous cell lung cancer has not been described so far. In the past, esophageal, lung, and mediastinal cancers were believed as the main malignancies responsible for acquired esophago-tracheobronchial fistula.^[6]^ Radiotherapy has also been described as a vital late cause.^[7]^

In the first case, the patient complained of shortness of breath and difficulty swallowing, which were signs of mediastinal metastasis invasion to the adjacent bronchus and esophagus. When the tumor shrunk significantly and rapidly after anlotinib treatment, an abnormal communication between esophagus and respiratory system formed. In the 2nd case, prior to anlotinib treatment, the patient has received multiple cycles of systemic chemotherapy and radiotherapy to the mediastinal lymph node metastasis, which highly likely have injured the tissue repair capacity of esophagus and bronchus. When the subcarinal lymph node metastasis shrunk greatly and rapidly after treatment (Fig. 2A and B), an esophago-tracheobronchial fistula developed probably due to previous injuries and inhibitory effect of anlotinib on angiogenesis. Both cases were diagnosed before the occurrence of pneumonia or mediastinitis because of the close attention to the early warning signs, which were immediately relieved after palliative treatment using fully covered self-expandable metallic stents.

Acquired esophago-tracheobronchial fistula is a clinically rare but life-threatening condition. Various causes contribute to the development of such an adverse event during anlotinib treatment. It should be suspected whenever patients with mediastinal metastasis develop warning clinical symptoms, for example, drinking water-induced choking cough. The diagnosis relies on esophagram, CT, and videofluoroscopy. For advanced lung cancer patients, the chance of surgical management is rather dim. Fully covered self-expandable metallic stent has been demonstrated as a superior alternative treatment owing to its high efficiency.^[6]^

### 3.1 Consent

Written informed consent was obtained from both patients for publication of this case report and any accompanying images. A copy of the written consent is available for review by the Editor-in-Chief of this journal.

## Author contributions

**Supervision:** Zengjun Liu.

**Writing – original draft:** Pinliang Zhang.

**Writing – review & editing:** Zengjun Liu.
